# T1ρ, T2 and T2* mapping of lumbar intervertebral disc degeneration: a comparison study

**DOI:** 10.1186/s12891-022-06040-y

**Published:** 2022-12-27

**Authors:** Li Yang, Cong Sun, Tao Gong, Quanlin Li, Xin Chen, Xinjuan Zhang

**Affiliations:** 1 Department of Radiology, Shanghai Institute of Medical Imaging, Zhongshan Hospital, Fudan University, Shanghai, 200032 People’s Republic of China; 2 Department of Radiology, Beijing Hospital, National Center of Gerontology, No. 1 Da-Hua Road, Dong Dan, Beijing, 100730 China; 3 Department of Radiology, Shandong Provincial Hospital Affiliated to Shandong First Medical University, Jinan, 250021 Shandong China; 4 Department of Radiology, Affiliated Hospital of Shandong University of Traditional Chinese Medicine, Jinan, 250012 Shandong China

**Keywords:** T1ρ mapping, T2 mapping, T2* mapping, Lumbar intervertebral disc, Degeneration

## Abstract

**Background:**

Early and accurate assessment of lumbar intervertebral disc degeneration (IVDD) is very important to therapeutic strategy. This study aims to correlate and compare the performances of T1ρ, T2 and T2* mapping for Pfirrmann grades and morphologic changes in the IVDD.

**Methods:**

This prospective study included 39 subjects with 195 lumbar discs. T1ρ, T2 and T2* mapping were performed, and T1ρ, T2 and T2* values of nucleus pulposus (NP), and anterior and posterior annulus fibrosus were measured. IVDD was assessed with Pfirrmann grading and morphologic changes (normal, bulging, herniation and annular fissure). The performances of T1ρ, T2 and T2* relaxation times were compared for detecting early (Pfirrmann grade II-III) and advanced degeneration (Pfirrmann grade IV–V), as well as for morphologic changes.

**Results:**

T2 relaxation times was strongly corelated with T1ρ and T2* relaxation times. Areas under the curves (AUCs) of T1ρ, T2 and T2* relaxation times of NP were 0.70, 0.87 and 0.80 for early degeneration, and 0.91, 0.95 and 0.82 for advanced degeneration, respectively. AUCs of T1ρ, T2 and T2* relaxation times of NP were 0.78, 0.83 and 0.64 for bulging discs, 0.87, 0.89 and 0.69 for herniated discs, and 0.79, 0.82 and 0.69 for annular tearing, respectively. The AUC of T2 relaxation time was significantly higher than those of T1ρ relaxation times (both *P* < 0.01) for early IVDD, and the AUCs of T1ρ and T2 relaxation times for assessing advanced degeneration and morphologic changes were similar (*P* > 0.05) but significantly higher than that of T2*relaxation time (*P* < 0.01).

**Conclusions:**

T2 mapping performed better than T1ρ mapping for the detection of early IVDD. T1ρ and T2 mapping performed similarly but better than T2* mapping for advanced degeneration and morphologic changes of IVDD.

**Supplementary Information:**

The online version contains supplementary material available at 10.1186/s12891-022-06040-y.

## Introduction

Low back pain affects up to 40%–80% of the population at some point during their lifetime, with considerable negative impacts on quality of life and social economy [1, 2]. Intervertebral disk degeneration (IVDD) is considered to be one of the primary causes of low back pain [1, 3]. Early phase of IVDD presents with biochemical changes, including decreases of overall proteoglycan and water content in the nucleus pulposus (NP). The later stages of IVDD are manifested in morphological changes, including disc bulging or herniation, annulus fibrosus (AF) tears, and vertebral osteophytes [4]. Unfortunately, current surgical treatments such as spinal fusion and replacement of disc are limited to pain relief treatment for severe IVDD. Surprisingly, recent studies showed regenerative strategies such as biologic and cell therapies may be applied for early-stage IVDD to limit disease progression [5, 6]. In this context, noninvasive imaging modalities are required for the detection of early changes and the severity of IVDD.

MRI is an important tool for evaluating IVDD. The Pfirrmann semi-quantitative classification is the most widely used for visual grading of IVDD [7], which is focused on the signal intensity and homogeneity of NP, distinction of the NP and AF, and height of the disc on sagittal T2-weighted images [8]. However, this scoring system is subjective, and it is unable to evaluate early biochemical changes of IVDD [9]. Besides the morphologic changes of disc bulging and herniation, high-intensity zone (HIZ) in the posterior annulus fibrosus (PAF) on T2-weighted images has been proposed to be an important sign for annular disruption and low back pain [10].

In recent years, various quantitative MRI have been applied to assess alterations of microenvironment of IVDD [11], such as T1ρ mapping [9, 12–14], T2 mapping [9, 15–17], T2* mapping and glycosaminoglycans chemical exchange saturation transfer [14, 18]. T1ρ mapping probes slow interactions between extracellular matrix macromolecules and bulk water by applying a “spin lock” pulse [19], while T2 and T2* relaxation times reflect the content and spatial distribution of macromolecule and water [19, 20]. It has been demonstrated that T1ρ, T2 and T2* relaxation times are associated with glycosaminoglycan and water content [13, 19, 20], Pfirrmann grading [11–13, 21–23], histological scores [17, 20, 21] and clinical symptom [24, 25] of IVDD. T1ρ is reported to be strong affinity with proteoglycan content in the disc matrix [13], and its main relaxation induced by chemical exchange [14]. T2* mapping is sensitive to collagen fiber network [26], while T2 mapping is more sensitive to tissue hydration, where dipolar interaction is the main relaxation mechanism [14].

Since T1ρ, T2 and T2* mapping have different mechanisms, thus they may have different sensitivity to compositional changes throughout IVDD [9], we hypothesized that these MRI parameters are fundamentally different when assessing disc health. However, no studies have been performed to evaluate the relative performance of T1ρ, T2 and T2* relaxation times for both lumbar disc degenerative grades and morphologic changes. The purpose of this study was to assess the correlation and compare the performance of T1ρ, T2 and T2* mapping for Pfirrmann grades and morphologic changes in the lumbar IVDD, thus to determine the most appropriate method for lumbar IVDD assessment.

## Material and methods

### Study population

The study was approved by the institute’s research ethics committee, and written informed consent was obtained from each participant prior to enrollment. Subjects with body mass index > 25, spinal fractures, tumors, infections, metabolic disease, previous lumbar disc surgery or interventional treatment, and contraindications for MR imaging were excluded. This study included 39 subjects (male 27, female 12; median age 44.0 years, age range 22–80 years) with single or recurrent episodes of low back pain and excluding other spine diseases except IVDD.

### MRI examinations

All subjects underwent MRI in the morning to reduce the potential impact of diurnal changes in the discs. Lumbar MRI including sagittal T2-weighted imaging, T1ρ mapping, T2 mapping, T2* mapping and axial T2-weighted imaging were performed at 3.0 T scanner (Achieva, Philips Healthcare, Best, the Netherlands) with a dedicated 15-channel SENSE spine coil. The imaging parameters are shown in Table 1. T1ρ-weighted images were obtained with a rotary echo spin-lock pulse embedded in a three-dimension balanced fast field echo sequence. Spin-lock frequency was set as 500 Hz, spin lock durations were 0, 10, 20, 30, 40 ms.

**Table 1:** Imaging parameters for T2-weighted imaging, T1ρ, T2 and T2* mapping

	sagittal T1ρ mapping	sagittal T2 mapping	sagittal T2^*^ mapping	sagittal T2-weighted images	axial T2-weighted images
field of view(mm^2^)	220 × 245	220 × 245	220 × 245	220 × 245	180 × 180
matrix	432 × 432	432 × 432	432 × 432	432 × 432	448 × 448
thickness	5 mm	5 mm	5 mm	5 mm	4 mm
slices	9	9	9	9	3/each disc
in-plane resolution	0.57 × 0.57	0.57 × 0.57	0.57 × 0.57	0.57 × 0.57	0.40 × 0.40
repetition time	4.8 ms	2000 ms	310 ms	2500 ms	3000 ms
echo time	2.4 ms	13/26/39/52 /65 ms	5.1/10.0/14.9/19.8/24.7 ms	90 ms	100 ms
flip angle	50°	90°	25°	90°	90°
NSA	1	1	2	1	1
scanning time	5 min 45 s	4 min 56 s	2 min 45 s	1 min 27 s	2 min 6 s

*NSA* Number of signal average

### Image analysis

A total of 195 discs covering L1–L2 to L5–S1 were analyzed. The morphological analyses were performed by the consensus of 2 experienced radiologist without knowledge of the quantitative MRI measurements. The assessments included Pfirrmann classifications on sagittal T2-weighted images [8], as well as the presence of disc bulging, herniation (protrusion or extrusion) according to the Lumbar Disc Nomenclature 2.0 [27], and annular fissure with HIZ of PAF on sagittal and axial T2-weighted images according to Aprill and Bogduk [9]. For Pfirrmann grades, IVDD with Pfirrmann grade I was normal disc, IVDD with Pfirrmann grade II-III were labeled as early degeneration, and IVDD with Pfirrmann grades IV and V were labeled as advanced degeneration [23].

**Fig. 1:**
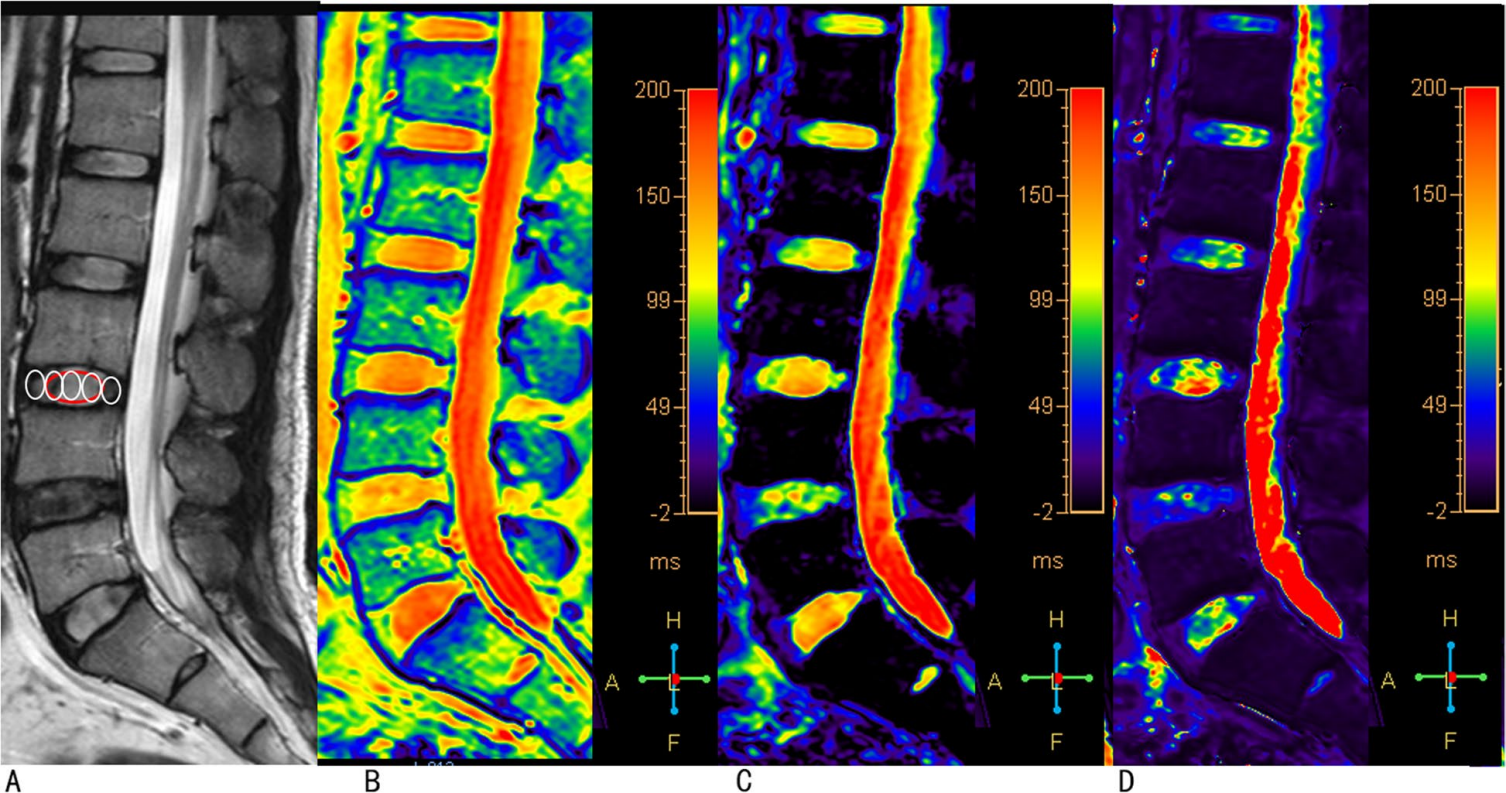
Representative images of sagittal T2-weighted images, T1ρ map, T2 map and T2* map. Five oval regions-of-interest (ROIs) were firstly placed on the T2-weighted images (**A**), then the ROIs were transferred to the T1ρ map (**B**), T2 map (**C**), and T2* map (**D**). The central three ovals (included in one red oval) represent nucleus pulposus, and the ventral and dorsal one represents anterior annulus fibrosus and posterior annulus fibrosus, respectively

The raw data of T1ρ mapping were fitted on a pixel-by-pixel basis to the exponentially decaying T1ρ function using IDL 6.3 (ITT Visual Information Solutions, Boulder, CO) to generate a T1ρ map, and the T2 and T2* maps were generated and analyzed by using Image J software (National Institutes of Health, Bethesda, MD). Regions of interest (ROIs) were manually placed on the NP, anterior annulus fibrosus (AAF) and PAF of the T1ρ, T2, and T2* maps (Fig. 1). The anterior and posterior 20% of the disk diameter were labeled as AF with the remaining central 60% representing the NP [23, 28]. Values of AAF and PAF were averaged as the value for the AF. All the ROI measurements were performed twice by an observer with an interval of one month.

### Statistical analysis

Numerical data were expressed as median and range as the data was not normally distributed according to the Shapiro—Wilk test. For the reliability of two measurements of T1ρ, T2 and T2* relaxation times, the intraclass correlation coefficients (ICCs) were calculated, and ICC values that were ≥ 0.75 were considered excellent agreement [29]. Wilcoxon test was used to compare the quantitative MRI parameters between NP and AF, as well as between discs with or without HIZ in the PAF. Pearson correlations were used to evaluate the relationship between every two quantitative MRI parameters, and between quantitative MRI parameters and subject age. Spearman rank correlations were applied to assess the association between quantitative MRI parameters and Pfirrmann grades, or morphology, as well as between subject age and Pfirrmann grades. The differences of quantitative MRI parameters in various Pfirrmann grades and morphology were evaluated with Kruskal–Wallis test and post-hoc tests. Furthermore, receiver operating characteristic (ROC) curves of each quantitative MRI parameter were plotted for detecting early IVDD (Pfirrmann grade II-III) and advanced IVDD (Pfirrmann grade IV–V), as well as for disc bulging or herniation, and annular tearing, and the cutoff values were defined with their sensitivities and specificities. The areas under the curves (AUCs) with 95% confidence intervals (CIs), sensitivities, and specificities were used to assess the diagnostic performances of quantitative MRI parameters. Delong method was used to compare the AUCs [30]. All statistical analyses were conducted using SPSS software (v23.0, IBM, Chicago, USA) and Medcalc (v18.2.1, Mariakerke, Belgium). A *P* value < 0.05 was regarded to be significantly different, and a Bonferroni-adjusted test was used for multiple comparisons.

## Results

### Measurements of T1ρ, T2 and T2* relaxation time

**Fig. 2:**
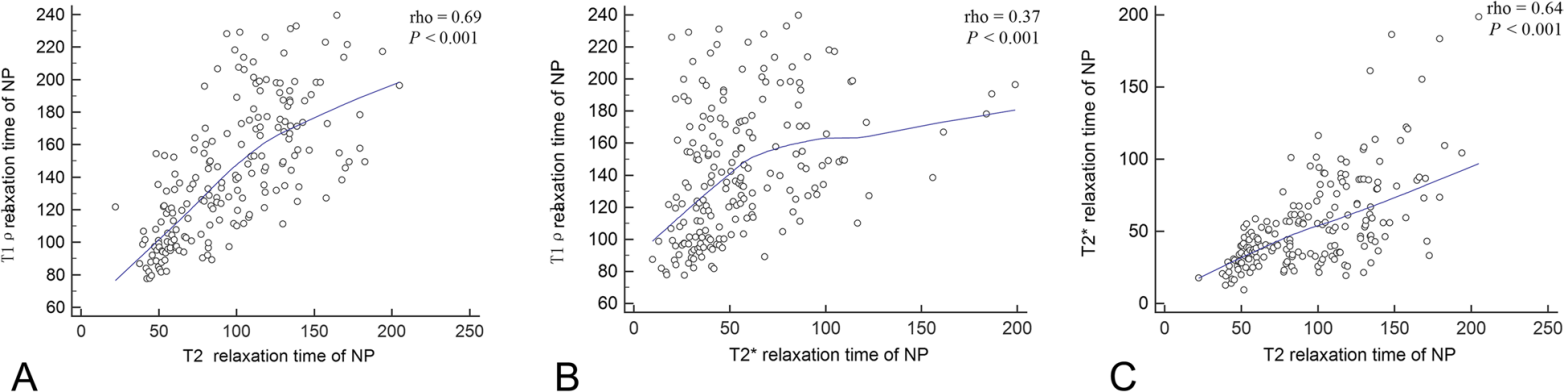
Correlations between T1ρ, T2 and T2* relaxation time of nucleus pulposus (NP). The T1ρ value in NP was significantly correlated with T2 (**A**) and T2* (**B**) values, and T2 values in NP was significantly correlated with T2* values (**C**)

The reproducibility between the two measurements for T1ρ, T2 and T2* relaxation times at different ROIs was excellent (ICCs = 0.88 ~ 0.93). The median values of NP and AF were 136.60 ms and 100.52 ms for T1ρ mapping, 93.53 ms and 36.99 ms for T2 mapping, and 46.05 ms and 21.18 ms for T2* mapping, respectively. There were significant differences in T1ρ, T2 and T2* values between NP and AF (all *P* < 0.001). The T1ρ value in NP was significantly correlated with T2 and T2* values (rho = 0.69, rho = 0.37, respectively, both *P* < 0.001, Fig. 2), and T2 values in both NP and AF were significantly correlated with T2* values (rho = 0.64, rho = 0.26, respectively, both *P* < 0.001), but no significant correlation was observed in AF between T1ρ and T2 values (*P* > 0.05), neither between T1ρ and T2* values (*P* > 0.05). The T1ρ, T2 and T2* values for NP, AAF and PAF at each disc level were not significantly correlated with subject age (Tables E1, E2 and E3), except T2* value of NP at level L2/3 (rho = -0.37, *P* = 0.02).

### T1ρ, T2 and T2* relaxation times for Pfirrmann grades

Of all 195 discs, the Pfirrmann grades were categorized as: I, 32 (16.4%) discs; II, 64 (32.8%) discs; III, 52 (26.7%) discs; IV, 40 (20.5%) discs; and V, 7 (3.6%) discs. The Pfirrmann grade was significantly corelated with subject age (rho = 0.60, *P* < 0.001).

**Fig. 3:**
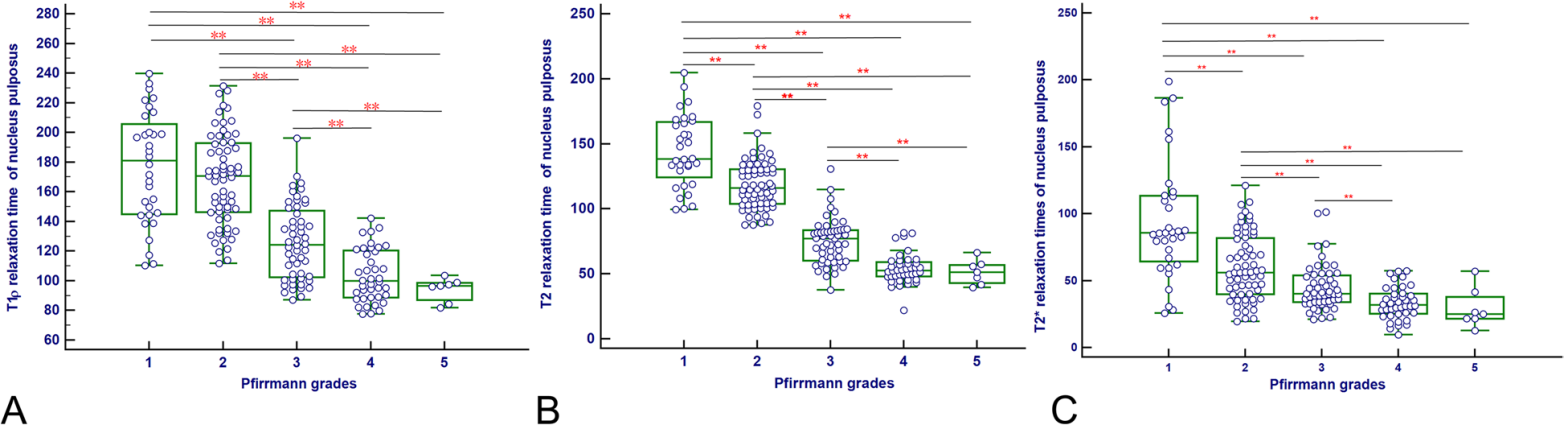
Box plots of the values in nucleus pulposus according to the Pfrrmann grades. **A**, **B** and **C** are T1ρ, T2 and T2* relaxation time of nucleus pulposus versus Pfrrmann grades, respectively. ** *P* < 0.005

T1ρ, T2 and T2* relaxation times of NP and AF were summarized in Table 2 and Table E4, and illustrated in Fig. 3. The T1ρ, T2 and T2* relaxation times of NP significantly decreased with the increase of Pfirrmann grades (rho = -0.73, *P* < 0.001; rho = -0.88, *P* < 0.001, rho = -0.61, *P* < 0.001; respectively).

**Table 2:** T1ρ, T2 and T2* relaxation times in nucleus pulposus for different Pfirrmann grades

Pfirrmann grades	T1ρ relaxation time (ms)	T2 relaxation time (ms)	T2* relaxation time (ms)
I (*n* = 32)	181.17 (110.23–239.88)	138.39 (99.48–204.70)	85.70 (25.81–198.85)
II (*n* = 64)	170.81 (111.61- 231.40)	114.98 (87.60–179.160)	56.28 (19.63- 121.16)
III (*n* = 52)	124.36 (87.11–196.19)	77.28 (37.55–130.76)	40.16 (20.97- 101.29)
IV (*n* = 40)	99.91 (77.69–142.08)	53.02 (22.02–146.67)	32.79 (14.27–57.33)
V (*n* = 7)	96.66 (81.80–103.62)	51.42 (39.52–66.45)	23.46 (9.62–53.21)
rho ^a^	-0.73	-0.88	-0.61
*P* value	< 0.001	< 0.001	< 0.001

Values are expressed as medians and ranges in parentheses

^a^Spearman rank correlations were applied to assess the association between quantitative MRI parameters and Pfirrmann grades

Regarding T1ρ relaxation times of NP, Kruskal–Wallis test and post-hoc tests showed significant differences between all pair-wise comparisons of Pfirrmann grades (all *P* < 0.005) except for between grades I and II and between grades IV and V (both *P* > 0.005). For T2 relaxation times in NP, Kruskal–Wallis test and post-hoc tests showed significant differences between all of the Pfrrmann grades (all *P* < 0.005) except between grades IV and V (*P* > 0.005). Kruskal–Wallis test and post-hoc tests of the T2* relaxation time of NP showed significant differences between all pair-wise comparisons of Pfirrmann grades (all *P* < 0.005) except for between III and V and between grades IV and V (both *P* > 0.05).

**Fig. 4:**
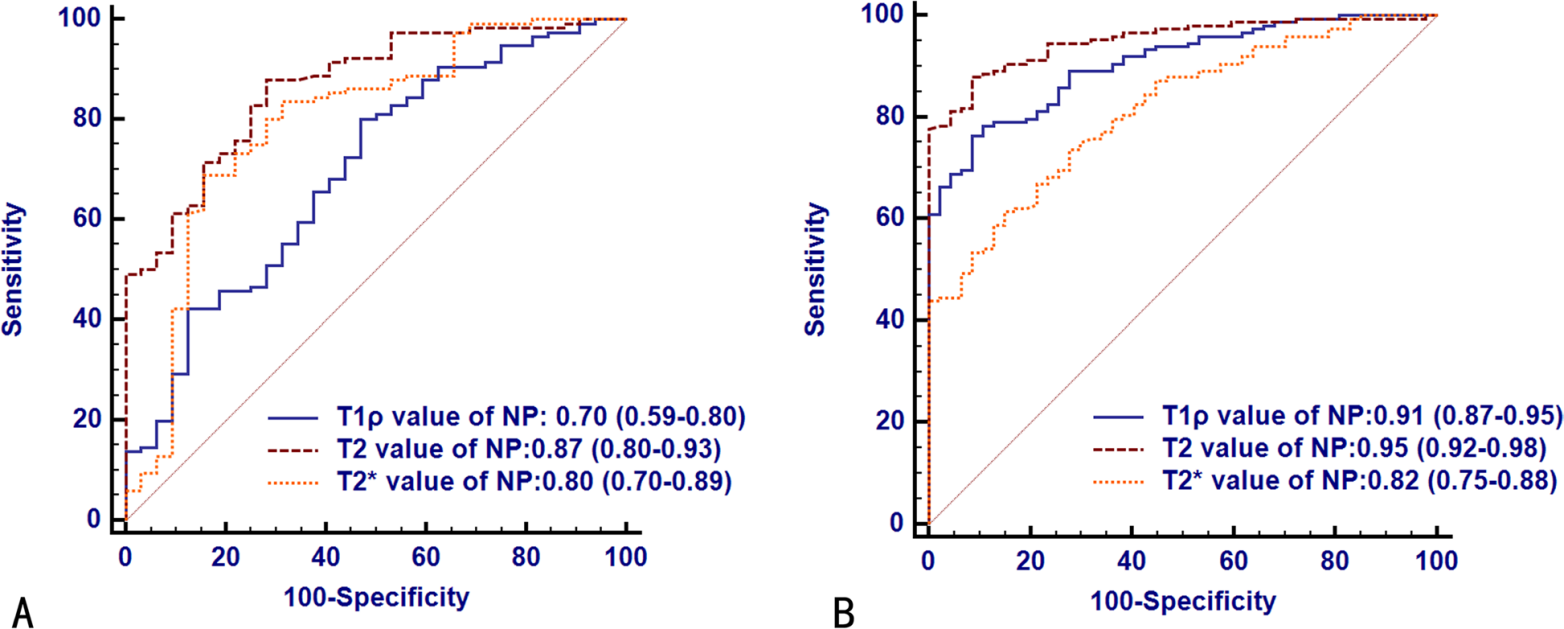
Receiver operating characteristic (ROC) analysis. Graphs show ROC curves of T1ρ, T2 and T2* mapping in nucleus pulposus (NP) for detecting early degeneration (Pfirrmann grade II-III, **A**) and advanced degeneration (Pfirrmann grade IV–V, **B**). Numbers are areas under the curves with 95% confidence intervals in parentheses

The ROC curves of T1ρ, T2 and T2* relaxation times for early and advanced IVDD are presented in Fig. 4 and the corresponding diagnostic performance are provided in Table 3 and Table E5. The AUCs of T1ρ, T2 and T2* relaxation times of NP were 0.70 (95%CI: 0.59-0.80), 0.87 (95%CI: 0.80-0.93) and 0.80 (95%CI: 0.70-0.89) for detecting early IVDD, and 0.91 (95%CI: 0.87-0.95), 0.95 (95%CI: 0.92-0.98) and 0.82 (95%CI: 0.75-0.88) for identifying advanced IVDD, respectively. For assessing early IVDD, the AUC of NP in T2 value was significantly higher than that in T1ρ value (*P* = 0.001), but the AUCs of NP in T2 and T1ρ value were not significantly different from that in T2* values (adjusted *P* > 0.05). For assessing advanced IVDD, the AUC of NP in T2 value was significantly higher than that in T2* values (*P* < 0.001), but the AUCs of T2 and T1ρ values did not showed significant difference (adjusted *P* > 0.05). The AUC of NP in T1ρ value was significantly higher than that in T2* values (*P* = 0.004).

**Table 3:** Diagnostic performance of T1ρ, T2 and T2* relaxation times of nucleus pulpous for different Pfirrmann grades

Modalities and Pfirrmann grades	AUC (95%CI)	*P* value	Cutoff (ms)	Sensitivity (%)	Specificity (%)
T1ρ mapping					
II -III *vs.* I	0.70 (0.59–0.80)	< 0.001	≤ 177.35	80.2	53.10
IV–V *vs.* I-III	0.91 (0.87–0.95)	< 0.001	≤ 125.35	91.49	76.35
T2 mapping					
II -III *vs.* I	0.87 (0.80–1.00)	< 0.001	≤ 131.14	87.93	71.87
IV–V vs. I-III	0.95 (0.92–0.98)	< 0.001	≤ 67.92	91.49	87.84
T2* mapping					
II- III *vs.* I	0.80 (0.70–0.89)	< 0.001	≤ 58.8	68.97	84.37
IV–V *vs.* I-III	0.82 (0.75–0.88)	< 0.001	≤ 45.68	85.11	61.49

### T1ρ, T2* and T2 relaxation times for disc bulging, herniation and annular tears

**Fig. 5:**
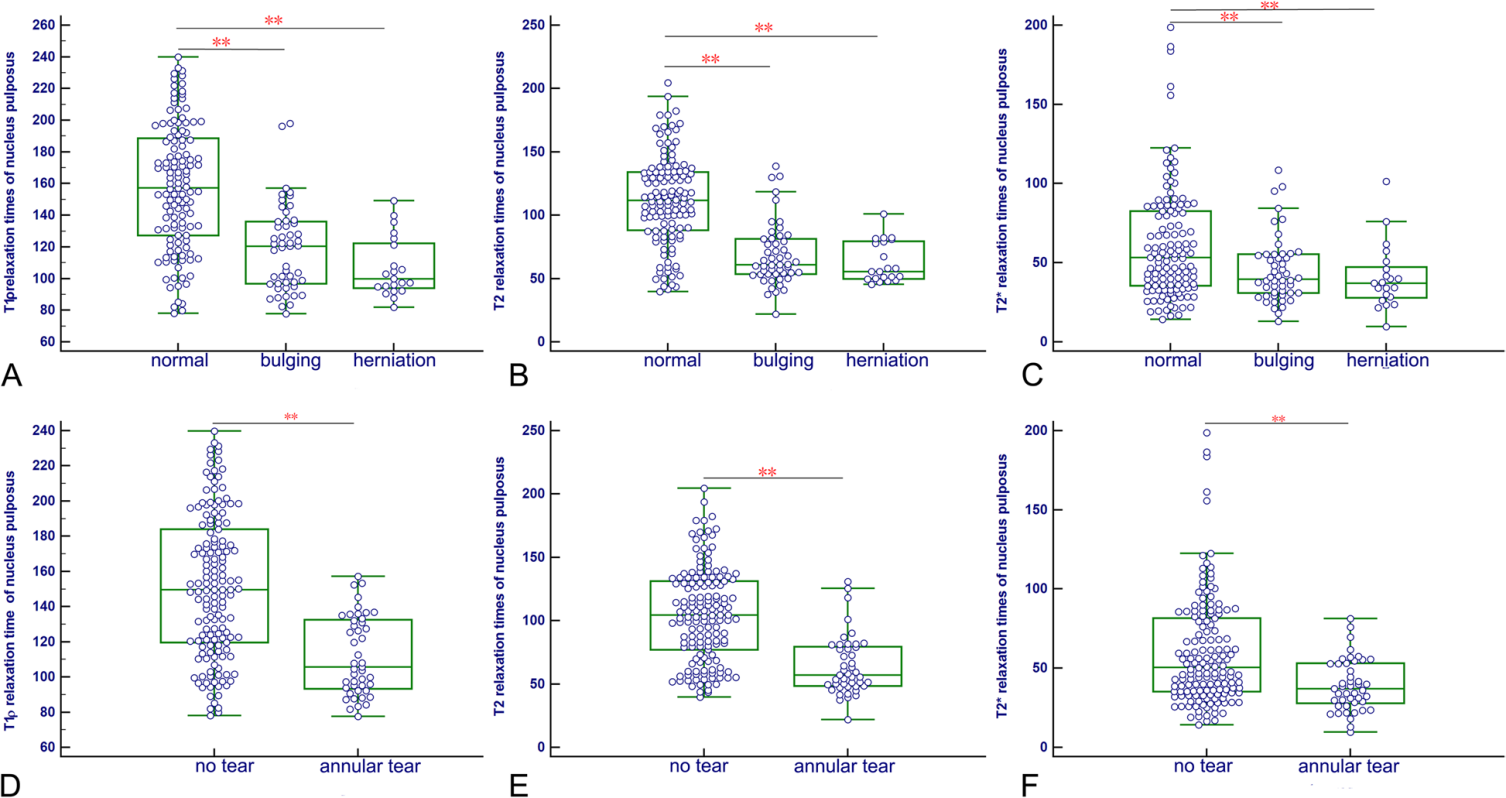
Box plots of the values in nucleus pulposus (NP) aaccording to the morphologic changes of disc degeneration. For comparisons of normal, bulging discs and herniated discs, **A**, **B** and **C** are respectively T1ρ, T2 and T2* relaxation time of NP. For comparison of no tear and annular tears, **D**, **E** and **F** are respectively T1ρ, T2 and T2* relaxation time of NP. ** *P* < 0.01

Of all 195 discs, 50 were labeled as bulging discs, 21 were herniated discs; 45 showed an HIZ in the PAF, reflecting annular tearing. T1ρ, T2 and T2* relaxation times of NP and AF for IVDD morphologic changes were showed in Table 4 and Table E6, and presented in Fig. 5. Kruskal–Wallis test and post-hoc tests showed that the T1ρ, T2 and T2* relaxation times of NP in normal discs were significantly higher than those in bulging or herniated discs and IVDD with HIZs in the PAF (*P* < 0.01), while there were no significant differences between bulging discs and herniated discs in all the MRI parameters (*P* > 0.05).

**Table 4:** T1ρ, T2 and T2* relaxation times in nucleus pulpous for lumbar disc bulging, herniation and annular tears

Morphologic changes	T1ρ relaxation time (ms)	T2 relaxation time (ms)	T2* relaxation time (ms)
Disc bulging or herniation			
Normal	157.43(77.96- 239.88)	111.69 (39.86–204.70)	53.41 (14.27–198.85)
Bulging	120.55(77.69–197.87)	61.07 (22.02–138.89)	39.69 (12.85–108.62)
Herniation	99.87 (81.80- 149.31)	55.68 (45.57–101.04)	37.17 (9.62–101.29)
rho^a^	-0.52	-0.58	-0.26
*P* vales	< 0.001	< 0.001	< 0.001
Annular tears			
No tear	149.67 (77.96–239.88)	104.47 (39.86–204.70)	50.58 (14.27–198.85)
High-intensity zones	105.73 (77.69–157.11)	57.18 (22.02–130.76)	37.17 (9.62–81.31)
*P* vales^b^	< 0.001	< 0.001	< 0.001

Values are expressed as medians and ranges in parentheses

^a^Spearman rank correlations were applied to assess the association between quantitative MRI parameters and morphologic changes

^b^Mann-Whitney U test was used to compare discs with and without annular tears

**Fig. 6:**
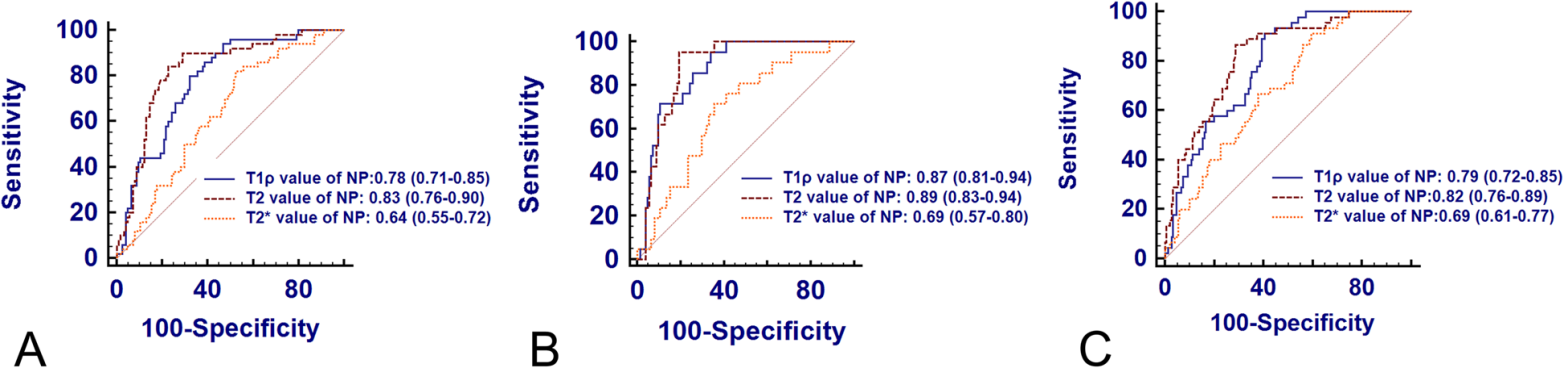
Receiver operating characteristic (ROC) analysis. Graphs show ROC curves of T1ρ, T2 and T2* mapping for detecting bulging discs (**A**), herniated discs (**B**), and annular tears (**C**). Numbers are areas under the curves with 95% confidence intervals in parentheses

The ROC curves of T1ρ, T2 and T2* relaxation times for morphologic changes of IVDD are presented in Fig. 6, and the corresponding diagnostic performance are provided in Table 5 and Table E7. Calculating a ROC for discriminating bulging discs from normal discs, the AUCs of T1ρ, T2 and T2* relaxation times of NP were 0.78 (95%CI: 0.71–0.85), 0.83 (95%CI: 0.76–0.90) and 0.64 (95%CI: 0.55–0.72), respectively. Moreover, the AUCs of T2 relaxation times was significantly higher than that of T1ρ relaxation time (*P* < 0.01), and the AUCs of T1ρ and T2 relaxation times were significantly higher than that of T2* relaxation time (*P* < 0.01).

**Table 5:** Diagnostic performance of T1ρ, T2 and T2* relaxation times in nucleus pulposus for lumbar disc bulging, herniation and annular tears

Modalities and morphologic changes	AUC (95%CI)	*P* value	Cutoff (ms)	Sensitivity (%)	Specificity (%)
T1ρ mapping					
Bulging *vs.* normal	0.78 (0.71–0.85)	< 0.001	≤ 136.94	80.00	67.74
Herniation *vs.* normal	0.87 (0.81–0.94)	< 0.001	≤ 139.88	95.24	66.13
annular tear *vs.* no tear	0.79 (0.72–0.85)	< 0.001	≤ 139.88	91.11	59.33
T2 mapping					
Bulging *vs.* normal	0.83 (0.76–0.90)	< 0.001	≤ 87.04	84.00	77.42
Herniation *vs.* normal	0.89 (0.83–0.94)	< 0.001	≤ 82.36	95.24	80.65
annular tear *vs.* no tear	0.82 (0.76–0.89)	< 0.001	≤ 82.28	86.67	71.33
T2* mapping					
Bulging *vs.* normal	0.64 (0.55–0.72)	0.002	≤ 55.85	82.00	47.58
Herniation *vs.* normal	0.69 (0.57–0.80)	0.009	≤ 41.66	71.43	64.52
annular tear *vs.* no tear	0.69 (0.61–0.77)	< 0.001	≤ 57.33	91.11	40.67

Calculating a ROC for discriminating herniated discs from normal discs, the AUCs of T1ρ, T2 and T2* relaxation times of NP were 0.87 (95%CI: 0.81–0.94), 0.89 (95%CI: 0.83–0.94) and 0.69 (95%CI: 0.57–0.80), respectively. Furthermore, the AUCs of T1ρ and T2 relaxation times were significantly higher than that of T2* relaxation time (*P* < 0.01).

Calculating a ROC for identifying HIZ of PAF from discs without tear, the AUC of T1ρ, T2 and T2* relaxation times of NP and T1ρ relaxation time of PAF were 0.79 (95%CI: 0.72–0.85), 0.82 (95%CI: 0.76–0.89) and 0.69 (95%CI: 0.61–0.77), respectively. In addition, the AUCs of T1ρ and T2 relaxation times of NP were significantly higher than that of T2* relaxation time (*P* < 0.01).

## Discussion

Our study demonstrated significantly moderate to strong negative correlations between the three MR quantitative parameters (T1ρ, T2 and T2* relaxation times) of NP and Pfirrmann grades, and T2 relaxation times was strongly corelated with T1ρ and T2* relaxation times. For identifying early IVDD (grade II-III), T2 mapping yielded greatest AUCs with high sensitivities, followed by T2* mapping and T1ρ mapping, indicating T2 mapping may be more accurate for the early disc degenerative changes. Additionally, T1ρ, T2 and T2* values of NP in normal discs differed significantly from those in bulging disc, herniated disc and annular tears. The AUCs of T1ρ and T2 mapping of NP were significantly higher than that of T2* mapping in detecting advanced IVDD (grade IV–V), bulging discs, herniated discs and annular tearing, implying T1ρ mapping and T2 mapping are more accurate than T2* mapping for late changes during IVDD.

Our present finding is consistent with previous studies that T1ρ, T2 and T2* relaxation times of NP decreased linearly with increasing Pfirrmann grades [11–13, 21–23]. Histologically, T1ρ is strong affinity with proteoglycan content in the disc matrix [13], T2 relaxation time has strong positive correlations with water and glycosaminoglycans content [19], and T2* relaxation time is highly correlated with glycosaminoglycan content [20]. During the IVDD process, the proteoglycan quantity diminishes and water content decreases [26], along with significant drop of T1ρ, T2 and T2* relaxation times of NP accordingly. T1ρ relaxation time of NP at Pfirrmann grade I was significantly higher than that at Pfirrmann grade III, IV and V, but there was no significant difference between grade I and II. However, there was significant differences in T2 and T2* relaxation times of NP between all pair-wise comparisons of Pfirrmann grades except for between grade IV and V for T2 and T2* mapping and between III and V for T2* mapping. The AUC of NP in T2 value was significantly higher than that in T1ρ value for assessing Pfirrmann grade II-III IVDD, with the cut-off values being similar to the Nagy et al. study [31]. This representation indicates T2-mapping may outperform the T1ρ mapping for detecting early stage IVDD. Yoon et al. reported T2 relaxation rates had stronger correlation with Pfirrmann grades than T1ρ value [9]. Menezes-Reis et al. [32] proposed that T2 mapping may be more appropriate than T1ρ mapping for the detection of early disc aging changes. Additionally, an ex vivo study showed T2 mapping was more sensitive to early degenerative changes than T1ρ mapping [19].

With regard to advanced degeneration and morphologic changes of IVDD (bulging discs, herniated discs and HIZs in the PAFs), the AUCs of NP in T2 and T1ρ mapping were similar (AUCs: 0.78–0.95) but significantly higher than that in T2* mapping (AUCs: 0.64–0.82). With ongoing IVDD, hydrophilic glycosaminoglycans within the NP is reduced or absent, result in structural defects such as annular tears and disc herniation [4]. Therefore, T2 and T1ρ mapping appeared to be sensitive to not only proteoglycan and water content but also collagen integrity. In line with this statement, a cadaveric study showed that the biomechanical properties of lumbar intervertebral discs are correlated with the collagen structure integrity [33].

Our results showed that T2 relaxation times was strongly correlated with T1ρ and T2* relaxation times. This may imply T2, T1ρ and T2* relaxation times are correlated to each other fundamentally, as all of them are associated with proteoglycan and water content [13, 19, 20]. A previous report also showed a significant linear association between T2 and T1ρ relaxation rates [9]. The results from our study suggest T2 mapping could be implemented as a synergistic modality with conventional MRI for assessing the severity of IVDD, which may help treatment option and prognosis evaluation of IVDD.

There are some limitations in present study. First, the number of patients included in this study was relatively small. However, power analysis depicted a minimum required number of 35 subjects with an alpha value of 0.05 and a specificity of 90%. Second, we only used Pfirrmann grade to assess IVDD, this grading system is subjective and cannot fully reflect the composition change during IVDD [33]. Meanwhile, histologic or biochemical validation of IVDD was lacking because we did not obtain specimens from the subjects. Further studies with humans and cadavers to validate the composition associated with different IVDD are warranted. Third, the echo times used in T2 mapping are relatively low, which may result in noise floor in T2 map, however the cut-off values of T2 mapping for early and advanced IVDD were similar to the Nagy et al. study [32]. Fourth, we did not correlate the T1ρ, T2 and T2* relaxation times with clinical symptom. Further study with a large cohort including clinical assessment is needed to clarify the relationship between MR quantitative parameters and clinical symptom.

## Conclusions

In conclusion, our resulted showed T2 mapping performed better than T1ρ mapping for the detection of early IVDD, and T1ρ and T2 mapping performed similarly but better than T2* mapping for advanced degeneration and morphologic changes of IVDD. T2 mapping may be of great utility for detecting the early and later changes of IVDD, as well as for monitoring the treatment response to emerging regeneration therapies.

## Supplementary Information

**Additional file 1: Table E1.** Correlation between subject age and T1ρ, T2* and T2 relaxation times of NP for each spinal level. **Table E2.** Correlation between age and T1ρ, T2* and T2 relaxation times of AAF for each spinal level. **Table E3.** Correlation between age and T1ρ, T2* and T2 relaxation times of PAF for each spinal level. **Table E4.** T1ρ, T2 and T2* relaxation times in AAF, PAF and AF for different Pfirrmann grades. **Table E5.** T1ρ, T2* and T2 relaxation times in AAF, PAF and AF for different Pfirrmann grades. **Table E6.** T1ρ, T2 and T2* relaxation times in AAF, PAF and AF for lumbar disc bulging, herniation and annular tears. **Table E7.** Diagnostic performance of T1ρ, T2 and T2* relaxation times in annulus fibrosus for lumbar disc bulging, herniation and annular tears.

## Data Availability

The datasets generated and/or analyzed during the current study are available from the corresponding author on reasonable request.

## Abbreviations

IVDD: Intervertebral disc degeneration
NP: Nucleus pulposus
AF: Annulus fibrosus
HIZ: High-intensity zone
PAF: Posterior annulus fibrosus
AAF: Anterior annulus fibrosus
TR: Repetition time
TE: Echo time
NSA: Number of signal average
ROIs: Regions of interest
ICCs: Intraclass correlation coefficients
CIs: Confidence intervals
ROC: Receiver operating characteristic
AUCs: Areas under the curves

## References

[1] Vlaeyen JWS, Maher CG, Wiech K, Van Zundert J, Meloto CB, Diatchenko L (2018). Low back pain. Nat Rev Dis Primers.

[2] Disease GBD, Injury I, Prevalence C (2018). Global, regional, and national incidence, prevalence, and years lived with disability for 354 diseases and injuries for 195 countries and territories, 1990–2017: a systematic analysis for the Global Burden of Disease Study 2017. Lancet (London, England).

[3] Brinjikji W, Diehn FE, Jarvik JG, Carr CM, Kallmes DF, Murad MH (2015). MRI findings of disc degeneration are more prevalent in adults with low back pain than in asymptomatic controls: a systematic review and meta-analysis. AJNR Am J Neuroradiol.

[4] Adams MA, Roughley PJ (2006). What is intervertebral disc degeneration, and what causes it?. Spine (Phila Pa 1976).

[5] Mohanty S, Dahia CL (2019). Defects in intervertebral disc and spine during development, degeneration, and pain: new research directions for disc regeneration and therapy. Wiley Interdiscip Rev Dev Biol.

[6] Binch ALA, Fitzgerald JC, Growney EA, Barry F (2021). Cell-based strategies for IVD repair: clinical progress and translational obstacles. Nat Rev Rheumatol.

[7] Gao F, Liu S, Zhang X, Wang X, Zhang J (2021). Automated grading of lumbar disc degeneration using a push-pull regularization network based on MRI. J Magn Reson Imaging.

[8] Pfirrmann CW, Metzdorf A, Zanetti M, Hodler J, Boos N (2001). Magnetic resonance classification of lumbar intervertebral disc degeneration. Spine (Phila Pa 1976).

[9] Yoon MA, Hong SJ, Kang CH, Ahn KS, Kim BH (2016). T1rho and T2 mapping of lumbar intervertebral disc: correlation with degeneration and morphologic changes in different disc regions. Magn Reson Imaging.

[10] Aprill C, Bogduk N (1992). High-intensity zone: a diagnostic sign of painful lumbar disc on magnetic resonance imaging. Br J Radiol.

[11] Tamagawa S, Sakai D, Nojiri H, Sato M, Ishijima M, Watanabe M (2022). Imaging evaluation of intervertebral disc degeneration and painful discs-advances and challenges in quantitative MRI. Diagnostics (Basel).

[12] Zhou Z, Jiang B, Zhou Z, Pan X, Sun H, Huang B (2013). Intervertebral disk degeneration: T1rho MR imaging of human and animal models. Radiology.

[13] Johannessen W, Auerbach JD, Wheaton AJ, Kurji A, Borthakur A, Reddy R (2006). Assessment of human disc degeneration and proteoglycan content using T1rho-weighted magnetic resonance imaging. Spine (Phila Pa 1976).

[14] Togao O, Hiwatashi A, Wada T, Yamashita K, Kikuchi K, Tokunaga C (2018). A Qualitative and quantitative correlation study of lumbar intervertebral disc degeneration using glycosaminoglycan chemical exchange saturation transfer, pfirrmann grade, and T1-rho. AJNR Am J Neuroradiol.

[15] Cao Y, Guo QW, Wan YD (2020). Significant association between the T2 values of vertebral cartilage endplates and pfirrmann grading. Orthop Surg.

[16] Li X, Xie Y, Lu R, Zhang Y, Li Q, Kober T (2022). Q-Dixon and GRAPPATINI T2 mapping parameters: a whole spinal assessment of the relationship between osteoporosis and intervertebral disc degeneration. J Magn Reson Imaging.

[17] Bouhsina N, Decante C, Hardel JB, Madec S, Abadie J, Hamel A (2021). Correlation between magnetic resonance, X-ray imaging alterations and histological changes in an ovine model of age-related disc degeneration. Eur Cell Mater..

[18] Pulickal T, Boos J, Konieczny M, Sawicki LM, Muller-Lutz A, Bittersohl B (2019). MRI identifies biochemical alterations of intervertebral discs in patients with low back pain and radiculopathy. Eur Radiol.

[19] Gullbrand SE, Ashinsky BG, Martin JT, Pickup S, Smith LJ, Mauck RL (2016). Correlations between quantitative T2 and T1rho MRI, mechanical properties and biochemical composition in a rabbit lumbar intervertebral disc degeneration model. J Orthop Res.

[20] Detiger SE, Holewijn RM, Hoogendoorn RJ, van Royen BJ, Helder MN, Berger FH (2015). MRI T2* mapping correlates with biochemistry and histology in intervertebral disc degeneration in a large animal model. Eur Spine J.

[21] Bouhsina N, Decante C, Hardel JB, Rouleau D, Abadie J, Hamel A (2022). Comparison of MRI T1, T2, and T2* mapping with histology for assessment of intervertebral disc degeneration in an ovine model. Sci Rep.

[22] Jiang Y, Yu L, Luo X, Lin Y, He B, Wu B (2020). Quantitative synthetic MRI for evaluation of the lumbar intervertebral disk degeneration in patients with chronic low back pain. Eur J Radiol.

[23] Wu LL, Liu LH, Rao SX, Wu PY, Zhou JJ (2022). Ultrashort time-to-echo T2* and T2* relaxometry for evaluation of lumbar disc degeneration: a comparative study. BMC Musculoskelet Disord.

[24] Blumenkrantz G, Zuo J, Li X, Kornak J, Link TM, Majumdar S (2010). In vivo 3.0-tesla magnetic resonance T1rho and T2 relaxation mapping in subjects with intervertebral disc degeneration and clinical symptoms. Magn Reson Med.

[25] Borthakur A, Maurer PM, Fenty M, Wang C, Berger R, Yoder J (2011). T1rho magnetic resonance imaging and discography pressure as novel biomarkers for disc degeneration and low back pain. Spine (Phila Pa 1976).

[26] Zeng F, Zha Y, Li L, Xing D, Gong W, Hu L (2019). A comparative study of diffusion kurtosis imaging and T2* mapping in quantitative detection of lumbar intervertebral disk degeneration. Eur Spine J.

[27] Fardon DF, Williams AL, Dohring EJ, Murtagh FR, Gabriel Rothman SL, Sze GK (2014). Lumbar disc nomenclature: version 2.0: recommendations of the combined task forces of the North American Spine Society, the American Society of Spine Radiology and the American Society of Neuroradiology. Spine J.

[28] Raudner M, Schreiner MM, Hilbert T, Kober T, Weber M, Szelenyi A (2021). Clinical implementation of accelerated T2 mapping: quantitative magnetic resonance imaging as a biomarker for annular tear and lumbar disc herniation. Eur Radiol.

[29] Shrout PE, Fleiss JL (1979). Intraclass correlations: Uses in assessing rater reliability. Psychol Bull.

[30] DeLong ER, DeLong DM, Clarke-Pearson DL. Comparing the areas under two or more correlated receiver operating characteristic curves: a nonparametric approach. Biometrics 1988;44(3):837–45. https://www.ncbi.nlm.nih.gov/pubmed/3203132. 3203132

[31] Nagy SA, Juhasz I, Komaromy H, Pozsar K, Zsigmond I, Perlaki G (2014). A statistical model for intervertebral disc degeneration: determination of the optimal T2 cut-off values. Clin Neuroradiol.

[32] Menezes-Reis R, Salmon CE, Bonugli GP, Mazoroski D, Tamashiro MH, Savarese LG (2016). Lumbar intervertebral discs T2 relaxometry and T1rho relaxometry correlation with age in asymptomatic young adults. Quant Imaging Med Surg..

[33] Schultz DS, Rodriguez AG, Hansma PK, Lotz JC (2009). Mechanical profiling of intervertebral discs. J Biomech.

