# Supplementary material for: T1ρ, T2 and T2* mapping of lumbar intervertebral disc degeneration: a comparison study
Source: BMC Musculoskelet Disord. 2022 Dec 27;23:1135. doi: 10.1186/s12891-022-06040-y (PMC9793566; doi:10.1186/s12891-022-06040-y)
Supplement: Supplementary file 1 — Additional file 1: Table E1. Correlation between subject age and T1ρ, T2* and T2 relaxation times of NP for each spinal level. Table E2. Correlation between age and T1ρ, T2* and T2 relaxation times of AAF for each spinal level. Table E3. Correlation between age and T1ρ, T2* and T2 relaxation times of PAF for each spinal level. Table E4. T1ρ, T2 and T2* relaxation times in AAF, PAF and AF for different Pfirrmann grades. Table E5. T1ρ, T2* and T2 relaxation times in AAF, PAF and AF for different Pfirrmann grades. Table E6. T1ρ, T2 and T2* relaxation times in AAF, PAF and AF for lumbar disc bulging, herniation and annular tears. Table E7. Diagnostic performance of T1ρ, T2 and T2* relaxation times in annulus fibrosus for lumbar disc bulging, herniation and annular tears. [file 12891_2022_6040_MOESM1_ESM.pdf]

Supplementary material

Correlation between subject age and T1ρ, T2* and T2 relaxation times of NP, and AF

The T1ρ, T2 and T2* values for both NP and AF regions throughout all levels were not significantly correlated with subject age (Table E1-E3), except T2* value of NP at level L2/3 (rho =-0.370, P =0.02).

Table E1 Correlation between subject age and T1ρ, T2* and T2 relaxation times of NP for each spinal level

Level	T1ρ relaxation time		T2 relaxation time		T2* relaxation time	
	Correlation coefficient	P value	Correlation coefficient	P value	Correlation coefficient	P value
L1/2	0.215	0.189	0.097	0.557	-0.172	0.294
L2/3	0.235	0.149	-0.093	0.574	**-0.370**	**0.020**
L3/4	0.305	0.059	-0.067	0.685	-0.160	0.330
L4/5	0.229	0.160	0.019	0.908	0.053	0.750
L5/S1	0.022	0.896	0.003	0.985	0.019	0.907

Table E2 Correlation between age and T1ρ, T2* and T2 relaxation times of AAF for each spinal level

Level	T1ρ relaxation time		T2 relaxation time		T2* relaxation time	
	Correlation coefficient	P value	Correlation coefficient	P value	Correlation coefficient	P value
L1/2	0.090	0.586	-0.092	0.577	-0.005	0.974
L2/3	0.243	0.137	0.143	0.386	0.075	0.650
L3/4	0.300	0.064	-0.023	0.892	-0.123	0.456
L4/5	0.123	0.455	-0.094	0.567	0.087	0.598
L5/S1	0.196	0.231	0.025	0.878	-0.134	0.415

Table E3 Correlation between age and T1ρ, T2* and T2 relaxation times of PAF for each spinal level

Level	T1ρ relaxation time		T2 relaxation time		T2* relaxation time	
	Correlation coefficient	P value	Correlation coefficient	P value	Correlation coefficient	P value
L1/2	0.153	0.352	0.114	0.488	0.196	0.233
L2/3	0.191	0.245	0.215	0.188	0.073	0.657
L3/4	0.193	0.240	0.061	0.713	0.133	0.419
L4/5	0.043	0.793	0.092	0.576	-0.159	0.333
L5/S1	0.140	0.395	-0.207	0.207	-0.134	0.415

T1ρ, T2 and T2* relaxation times of AAF, PAF and AF for Pfirrmann grades

T1ρ, T2 and T2* relaxation times of AF were summarized in Table E4. The T1ρ relaxation times of AAF, PAF and AF had weak correlations with Pfirrmann grades (rho = -0.305, rho = -0.314, rho =-0.356, $P < 0.001$, respectively), as well as the T2 relaxation times of PAF and AF (rho = 0.224, rho = 0.156, $P < 0.05$, respectively), but the T2 relaxation times of AAF and T2* relaxation time of AAF, PAF and AF had no correlation with Pfirrmann grades ($P > 0.05$). For the T1ρ relaxation times of AF, Pfirrmann grade II was significantly different from Pfirrmann grades higher than II (all $P < 0.005$), and there were significant differences between grades I and IV and between grades I and V (both $P < 0.005$), while no significant differences were found between grades I and II, between I and grades III, and between grades IV and V (all $P > 0.005$). For the AF, Kruskal-Wallis test showed no significant difference in T2 values among different Pfirrmann grades ($P > 0.05$). For the T2* values of AF, Kruskal-Wallis test showed a marginal difference among different Pfirrmann grades ($P = 0.049$), but no significant differences were found between all pair-wise comparisons of Pfirrmann grades (all $P > 0.005$).

The diagnostic performance of T1ρ, T2 and T2* relaxation times of AAF, PAF and AF for Pfirrmann grade III and Pfirrmann grade IV–V IVDD are provided in Table E5. The AUCs of T1ρ, relaxation times of AAF, PAF and AF and T2 relaxation times of AAF and AF were 0.62 (95%CI: 0.53-0.72), 0.66 (95%CI: 0.56-0.76), 0.67 (95%CI: 0.58-0.77), 0.63 (95%CI: 0.54-0.73) and 0.61 (95%CI: 0.52-0.71) for detecting Pfirrmann grade III IVDD. The AUCs of T1ρ relaxation times of AAF, PAF and AF and T2 relaxation times of PAF were for 0.70 (95%CI: 0.62-0.78), 0.68 (95%CI: 0.59-

0.76), 0.71 (95%CI: 0.62-0.80) and 0.64 (95%CI: 0.55-0.74) for identifying Pfirrmann grade IV–V IVDD, respectively. There were no significant differences of these MR parameters for different grades fo IVDD (all P > 0.05).

Table E4 T1ρ, T2 and T2* relaxation times in AAF, PAF and AF for different Pfirrmann grades

Pfirrmann grades	T1ρ relaxation time (ms)			T2 relaxation time (ms)			T2* relaxation time (ms)		
	AAF	PAF	AF	AAF	PAF	AF	AAF	PAF	AF
Ⅰ (n=32)	99.09 (51.63-170.75)	109.27 (73.89-183.15)	103.00 (76.84-176.05)	36.49 (24.98-63.78)	34.67 (26.97-43.42)	35.22 (28.63-49.11)	23.16 (10.18-42.47)	22.68 (13.17-33.11)	23.76 (14.36-34.99)
Ⅱ (n=64)	108.29 (61.54-179.88)	115.67 (67.15-189.71)	112.85 (82.88-183.49)	36.02 (18.74-53.12)	35.70 (23.11-59.63)	35.97 (20.93-47.72)	20.14 (9.66-36.90)	18.40 (9.63-39.74)	19.32 (12.79-33.98)
Ⅲ (n=52)	93.65 (62.13-146.66)	97.20 (61.13-160.92)	96.03 (71.27-153.79)	40.30 (25.51-58.78)	37.06 (26.11-47.14)	39.00 (25.93-48.40)	20.52 (12.72-38.73)	20.97 (11.26-54.84)	21.30 (12.61-37.53)
Ⅳ (n=40)	87.48 (67.63-135.18)	98.07 (70.04-144.72)	92.03 (72.10-136.33)	35.59 (27.99-62.35)	39.37 (24.24-57.57)	37.71 (27.56-53.11)	20.91 (11.72-38.78)	20.45 (10.35-42.82)	21.54 (11.95-34.80)
Ⅴ (n=7)	75.86 (55.21-101.42)	84.80 (55.23-138.97)	79.98 (55.22-117.82)	34.46 (19.01-42.59)	39.19 (34.20-51.00)	37.59 (32.74-42.37)	18.86 (11.85-26.77)	19.96 (14.55-42.82)	20.00 (13.54-29.69)
rho[a]	-0.305	-0.314	-0.356	0.029	0.224	0.152	-0.057	0.014	-0.007
P vales	<0.001	<0.001	<0.001	0.687	0.002	0.034	0.431	0.846	0.925

Note—AAF= anterior annulus fibrosus, PAF= posterior annulus fibrosus, AF=annulus fibrosus. Values are expressed as medians and ranges in parentheses.

[a]Spearman rank correlations were applied to assess the association between quantitative MRI parameters and Pfirrmann grades

Table E5 T1ρ, T2* and T2 relaxation times in AAF, PAF and AF for different Pfirrmann grades

Modalities and Pfirrmann grades	AUC (95%CI)	P value	Cutoff (ms)	Sensitivity (%)	Specificity (%)
T1ρ mapping of AAF					
III *vs*. I-II	0.62 (0.53-0.72)	0.01	≤97.62	63.46	60.42
IV–V *vs*. I-III	0.70 (0.62-0.78)	<0.001	≤90.84	65.96	70.27
T1ρ mapping of PAF					
III *vs*. I-II	0.66 (0.56-0.76)	0.001	≤99.38	55.77	78.12
IV–V *vs*. I-III	0.68 (0.59-0.76)	<0.001	≤109.22	80.85	50.68
T1ρ mapping of AF					
III *vs*. I-II	0.67 (0.58-0.77)	<0.001	≤100.89	63.46	65.62
IV–V *vs*. I-III	0.71 (0.62-0.80)	<0.001	≤95.09	65.96	66.89
T2 mapping of AAF					
III *vs*. I-II	0.63 (0.54-0.73)	0.006	>40.15	53.85	75.00
T2 mapping of PAF					
IV–V *vs*. I-III	0.64 (0.55-0.74)	0.003	>38.63	55.32	69.59
T2 mapping of AF					
III *vs*. I-II	0.61 (0.52-0.71)	0.02	>38.105	55.77	67.71

Note—AAF= anterior annulus fibrosus, PAF= posterior annulus fibrosus, AF=annulus fibrosus.

T1ρ, T2* and T2 relaxation times for disc bulging, herniation and annular tears

T1ρ, T2 and T2* relaxation times in AAF, PAF and AF for lumbar disc bulging, herniation and annular tears are shown in Table E6. Kruskal-Wallis test and post-hoc tests showed that the T1ρ relaxation times of PAF and AF in normal discs were significantly higher than those in bulging or herniated discs and IVDD with HIZs in the PAF ($P \leq 0.01$), while T1ρ relaxation times of AAF, and T2 and T2* relaxation times of AAF, PAF and AF in normal discs did not differ significantly from those in bulging or herniated discs and IVDD with HIZs (all $P > 0.05$). There were no significant differences between bulging discs and herniated discs in all the MRI parameters (all $P > 0.05$).

Diagnostic performance of T1ρ, T2 and T2* relaxation times in annulus fibrosus for lumbar disc bulging, herniation and annular tears are shown in Table E7. There were no significant differences between each MR parameters (all P>0.05).

Table E6 T1ρ, T2 and T2* relaxation times in AAF, PAF and AF for lumbar disc bulging, herniation and annular tears

Morphologic	T1ρ relaxation time (ms)			T2 relaxation time (ms)			T2* relaxation time (ms)		
changes	AAF	PAF	AF	AAF	PAF	AF	AAF	PAF	AF
Disc bulging or herniation									
Normal	99.74 (51.63-179.88)	110.86 (67.15-189.71)	104.38 (73.24-183.49)	36.14 (18.74-63.78)	35.98 (23.11-59.63)	36.27 (20.93-49.11)	20.40 (9.66-42.47)	19.05 (9.63-39.74)	20.19 (11.95-34.99)
Bulging	93.23 (55.21-146.66)	98.31 (55.23-160.92)	94.52 (55.22-153.79)	36.50 (19.01-58.780)	38.37 (24.08-57.57)	38.31 (25.93-48.40)	20.88 (11.85-38.78)	22.11 (10.35-42.82)	23.52 (13.54-34.95)
Herniation	90.65 (67.31-122.01)	89.30 (61.13-131.77)	91.39 (70.76-133.64)	40.93 (28.44-62.35)	37.52 (29.08-50.27)	39.24 (29.98-53.11)	21.73 (12.72-38.24)	18.90 (13.35-54.84)	22.43 (13.37-37.53)
rho[a]	-0.20	-0.27	-0.28	0.15	0.17	0.20	0.12	0.11	0.17
P vales	0.005	0.001	<0.001	0.03	0.02	0.006	0.10	0.13	0.02
Annular tears									
No tear	97.02 (51.63-179.88)	108.55 (70.55-189.71)	101.66 (73.24-183.49)	36.54 (18.74-63.78)	36.26 (23.11-59.63)	36.67 (20.93-49.11)	20.30 (9.66-42.47)	19.41 (9.63-54.84)	20.55 (11.95-37.53)
HIZs	93.86 (55.21-130.43)	98.58 (55.23-148.98)	94.39 (55.22-136.33)	36.36 (19.01-62.35)	38.51 (26.11-51.00)	38.49 (25.93-53.11)	21.54 (11.85-38.24)	20.41 (12.10-42.82)	22.43 (13.37-35.46)
P vales[b]	0.07	<0.01	0.01	0.40	0.08	0.12	0.46	0.30	0.12

Note—AAF= anterior annulus fibrosus, PAF= posterior annulus fibrosus, AF=annulus fibrosus, HIZs= High-intensity zones

Values are expressed as medians and ranges in parentheses.

[a]Spearman rank correlations were applied to assess the association between quantitative MRI parameters and morphologic changes

[b]Mann-Whitney U test was used to compare discs with and without annular tears

Table E7 Diagnostic performance of T1ρ, T2 and T2* relaxation times in annulus fibrosus for lumbar disc bulging, herniation and annular tears

Modalities and morphologic changes	AUC (95%CI)	P value	Cutoff (ms)	Sensitivity (%)	Specificity (%)
T1ρ mapping of PAF					
Bulging *vs*. normal	0.63 (0.53-0.72)	0.008	≤109.22	72.00	53.23
Herniation *vs*. normal	0.73 (0.61-0.85)	<0.001	≤107.02	80.95	55.65
annular tear *vs*. no tear	0.63 (0.54-0.73)	0.006	≤111.83	77.78	44.67
T2 mapping of PAF					
Bulging *vs*. normal	0.62 (0.52-0.71)	0.014	>36.44	64.00	55.65
T2* mapping of PAF					
Bulging *vs*. normal	0.61 (0.52-0.71)	0.020	>19.29	70.00	52.42
T1ρ mapping of AF					
Bulging *vs*. normal	0.62 (0.53-0.72)	0.008	≤95.09	56.00	67.74
Herniation *vs*. normal	0.72 (0.60-0.84)	0.002	≤112.3	95.24	40.32
annular tear *vs*. no tear	0.62 (0.53-0.71	0.006	≤105.14	77.78	46.00
T2 mapping of AF					
Bulging *vs*. normal	0.61(0.52-0.70)	0.018	>35.57	74.00	45.97
Herniation *vs*. normal	0.64 (0.51-0.77)	0.037	>35.79	76.19	47.58
T2* mapping of AF					
Bulging *vs*. normal	0.63 (0.53-0.72)	0.008	>24.13	46.00	78.23
